# Efficacy and Safety of Syk and BTK Inhibitors in Immune Thrombocytopenia: A Comprehensive Review of Emerging Evidence

**DOI:** 10.1155/mi/5578929

**Published:** 2025-05-09

**Authors:** Amirhossein Heidari, Amirhossein Shahbazi Mazid, Mohammad Behroozfar, Negar Ghotbi, Fatemeh Fathabadi, Sara Eghbali, Nazila Heidari

**Affiliations:** ^1^Faculty of Medicine, Tehran Medical Sciences, Islamic Azad University, Tehran, Iran; ^2^Cardiovascular Diseases Research Institute, Research Center for Advanced Technologies in Cardiovascular Medicine, Tehran University of Medical Sciences, Tehran, Iran; ^3^Department of Immunology, Faculty of Medicine, University of Debrecen, Debrecen 4032, Hungary; ^4^School of Medicine, Tehran University of Medical Sciences, Tehran, Iran; ^5^School of Medicine, Guilan University of Medical Sciences, Rasht, Iran; ^6^School of Medicine, Iran University of Medical Sciences, Tehran, Iran

**Keywords:** Bruton's tyrosine kinase, immune thrombocytopenia, ITP, spleen tyrosine kinase

## Abstract

Immune thrombocytopenia (ITP) is an autoimmune disorder characterized by a reduced platelet count, resulting in bleeding risks and compromised quality of life. Advances in understanding ITP pathogenesis have revealed critical roles for spleen tyrosine kinase (Syk) and Bruton's tyrosine kinase (BTK) in Fc receptor (FcR)-mediated immune pathways, which are central to autoantibody production and platelet destruction. We sought to evaluate the efficacy and safety of Syk and BTK inhibitors in the management of ITP. PubMed/Medline, Scopus, and Web of Science databases were systematically searched up to July 28, 2024. Clinical studies with available full-text in English were included. Fostamatinib, an FDA-approved Syk inhibitor, has shown efficacy in enhancing platelet counts and reducing bleeding events in refractory ITP patients. Among the newer Syk inhibitors, sovleplenib demonstrated rapid and sustained platelet increases in clinical trials, with an 80% response rate at the 300 mg dosage and a favorable safety profile. Additionally, BTK inhibitors, including rilzabrutinib and orelabrutinib, have shown potential in clinical trials, offering increased platelet stability and favorable safety profiles in ITP cases. Syk and BTK inhibitors hold potential as targeted therapies for refractory ITP, with evidence supporting their ability to improve clinical outcomes and enhance patient quality of life. Continued research is warranted to optimize these therapies and confirm their long-term efficacy and safety in diverse patient populations.

**Summary**

• Immune thrombocytopenia (ITP) treatments pose substantial challenges, particularly for refractory cases, impacting patients' quality of life and healthcare utilization.
• Spleen tyrosine kinase (Syk) and Bruton's tyrosine kinase (BTK) are key mediators in Fc receptor (FcR)-driven immune responses that contribute to platelet destruction and autoantibody production in ITP.
• Targeted therapies such as fostamatinib, sovleplenib, rilzabrutinib, and orelabrutinib have shown promising results in enhancing platelet counts and stabilizing outcomes in patients with refractory ITP who failed first and second treatment lines.

## 1. Introduction

Immune thrombocytopenia (ITP) is an acquired disorder of thrombocytopenia, marked by autoimmune destruction and decreased production of platelets, leading to a reduction in peripheral blood platelet count [1]. The prevalence of ITP is 9.5 per 100,000 adults and the incidence is 2–5 per 100,000 adults per year [2, 3]. ITP can be classified into two types: primary ITP, which is idiopathic and diagnosed by excluding other potential causes, and secondary ITP, which is associated with underlying conditions such as infections, systemic lupus erythematosus, *Helicobacter pylori* infection, or hematologic malignancies [4, 5]. Thrombocytopenia enhances the risk of bleeding; while many individuals with ITP remain asymptomatic, a considerable proportion experience mucocutaneous bleeding, ranging from mild manifestations, such as petechiae, purpura, and epistaxis, to more severe and life-threatening complications, including gastrointestinal or intracranial hemorrhage [6, 7]. Further, ITP has been suggested to increase the incidence of thromboembolic events in various studies [8, 9]. The incidence of arterial thrombosis in patients with ITP is reported to range from 9.6 to 27.8 per 1000 person per year, which is slightly higher than the incidence observed in the general population [10]. Moreover, among patients with ITP, the incidence of venous thromboembolism is projected to range from 4.1 to 6.7 per 1000 person per year, reflecting approximately twice the risk observed in the general population, as reported by most studies. The condition also negatively impacts health-related quality of life through fatigue, limitations in daily activities, anxiety, and other associated sequelae [11, 12].

Recent research has shown that aberration in both humoral and cellular immunity plays a role in the pathophysiology of ITP [13]. Dendritic cells and CD4+ regulatory T cells are involved in both the initiation and maintenance of ITP. Patients with active disease have been reported to have decreased CD4+ CD25+ FOXP3+ T regulatory cell levels and function. Additionally, autoantibodies facilitate the recognition of platelets by macrophages expressing Fc*γ* receptors (Fc*γ*Rs), resulting in their destruction through phagocytosis [14]. Cytotoxic CD8+ T cells can cause thrombocytopenia by directly lysing platelets [15]. Autoantibodies or CD8+ T cells targeting megakaryocytes (MKs) in the bone marrow led to functional impairment and reduced platelet production [16]. In the process of B-cell activation and autoantibody production, the spleen tyrosine kinase (Syk) plays a critical role, which is activated through a series of biochemical reactions by immune cell receptors, such as Fc*γ*Rs, B-cell receptors (BCRs), and natural killer cell receptors. Fc*γ*R signaling mediates platelet phagocytosis, so Syk represents a potential therapeutic target for the treatment of ITP [17, 18]. Additionally, Bruton's tyrosine kinase (BTK) is another essential molecule involved in B-cell antigen receptor signaling necessary for B-cell proliferation and differentiation, leading to immune responses and the production of cytokines [19, 20]. As a result of blocking BTK signaling, BTK inhibitors can potentially affect autoimmune diseases involving B cells and non-B cells [20].

In the treatment of ITP, the goal involves raising the platelet count to prevent further bleeding, stop life-threatening active bleeding, and improve patients' quality of life [21, 22]. Glucocorticoids (most commonly oral prednisolone or prednisone) as immune response modulators are the standard initial treatment for acute ITP. Steroids inhibit autoantibody production and downregulate macrophage activity, which is responsible for platelet phagocytosis. Corticosteroid therapy is associated with hyperglycemia, weight gain, osteoporosis, and gastric irritation, which may be difficult to tolerate for some patients [3, 22]. Intravenous immunoglobulin (IVIG) is the other mainstay of medical therapy for treatment due to its anti-inflammatory and immunomodulatory effects [23]. It can be used in emergencies of thrombocytopenia active bleeding or when an urgent invasive procedure is needed. The most common side effects of IVIG include headache, hypertension, allergic reactions, and some more serious adverse effects, such as anaphylaxis in IgA-deficient individuals, hemolytic anemia, acute kidney injury, thrombosis, and transmission of bloodstream infections [24]. Anti-D immunoglobulin, which is composed of immunoglobulin G and directed against the D antigen of the Rh blood group system, can be an effective option as first-line therapy for rapidly increasing platelet counts in ITP [25]. Intravenous infusion of anti-D immunoglobulin into a D-positive recipient results in antibody coating of circulating RBCs, which are cleared primarily by the spleen [26]. Most commonly, IVIG-like reactions occur during the infusion of anti-D immunoglobulin and it can also cause extravascular hemolysis, resulting in hemolytic anemia and acute hemolytic transfusion reaction, making it less preferred than IVIG [27, 28]. Treatment options used in second-line therapy, including thrombopoietin receptor agonists (TPO-RAs), splenectomy, and rituximab, are beneficial for patients who cannot maintain long-term safe platelet count and show relapse later during life [3]. Syk-signaling pathway, as mentioned before, has emerged as a potential target for the treatment of ITP. Fostamatinib, the only licensed Syk inhibitor to date, produces clinical responses in those patients who are refractory to other therapies [29]. Rilzabrutinib is an oral, reversible, and potent BTK inhibitor explicitly designed to treat immune-mediated diseases such as ITP [30]. There are also some other non-FDA-approved tyrosine kinase (Tyk) inhibitors that demonstrate potential in the management of ITP. In this review, we aim to review the role of the Tyk signaling pathway in the development of ITP and focus on novel non-FDA-approved drugs that are used in ITP treatment.

## 2. Methods

**Figure 1:**
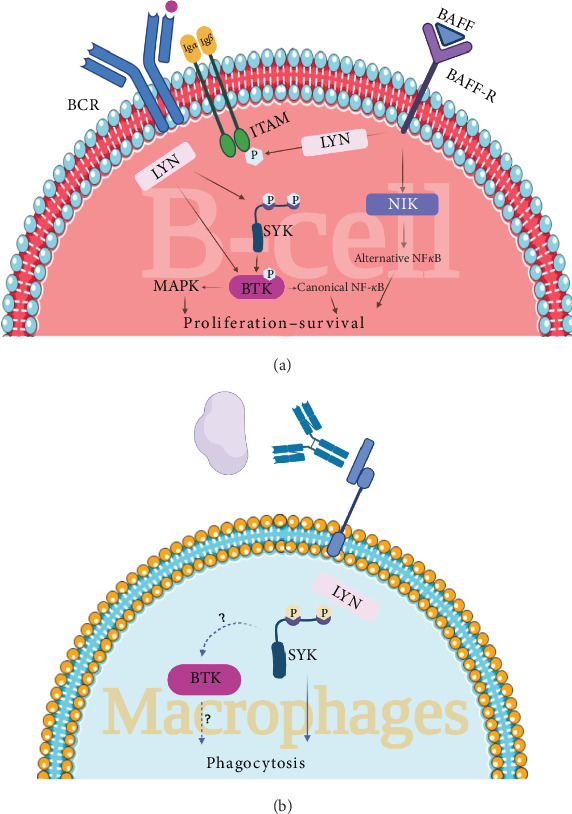
The mechanism of action of spleen tyrosine kinase (Syk) and Bruton tyrosine kinase (BTK) inhibitors in B-cells (A) and macrophages (B) in the treatment of immune thrombocytopenia (ITP).

Web of Science, Scopus, and PubMed/Medline databases were systematically searched using keywords and MESH terms for studies published up to July 28, 2024. Search terms used for the search were as follow: “Bruton's tyrosine kinase inhibitor*⁣*^*∗*^” OR “Bruton's tyrosine kinase” OR “BTK inhibitor*⁣*^*∗*^” OR “BTKI” OR “Bruton tyrosine kinase inhibitor*⁣*^*∗*^” OR “Bruton tyrosine kinase” OR “ibrutinib” OR “Imbruvica” OR “acalabrutinib” OR “Calquence” OR “zanubrutinib” OR “Brukinsa” OR “pirtobrutinib” OR “Jaypirca” OR “tirabrutinib” OR “rilzabrutinib” OR “remibrutinib” OR “Evobrutinib” OR “Spebrutinib” OR “Vecabrutinib” OR “pirtobrutinib” OR “orelabrutinib” OR “branebrutinib” OR “fenebrutinib” OR “tolebrutinib” OR “fostamatinib” OR “sovleplenib” OR “Entospletinib” OR “Cerdulatinib” OR “Syk Kinase inhibitor*⁣*^*∗*^” OR “Cevidoplenib” OR “Syk Tyrosine Kinase inhibitor*⁣*^*∗*^” OR “Spleen Tyrosine Kinase inhibitor*⁣*^*∗*^” AND “Autoimmune thrombocytopenia” OR “Immune thrombocytopenia” OR “Immune thrombocytopenic purpura” OR “ITP.” The clinical studies (clinical trials, observational studies, case series, and case reports) with full text published in English were included in this review. As a result, a total of five published clinical studies were included. Tables 1 and 2 comprehensively demonstrate the characteristics of the non-FDA-approved Syk and BTK inhibitor medications that have been investigated in ITP patients, respectively. Figure (1A,B) illustrates the mechanism of action of Syk and BTK in the pathogenesis of ITP in B-cells and macrophages, respectively.

## 3. Result and Discussion

### 3.1. The Role of Tyk in the Pathophysiology of ITP

The exact underlying mechanism of ITP has not been elucidated yet; however, pathological antibody-mediated platelet clearance [34], T cell-mediated platelet destruction [15], and impaired function of MK are the three major known mechanisms in ITP development [6]. The primary clearance site for antibody-coated platelets is the spleen, where they bind to tissue macrophages through Fc receptors (FcRs) [34] and this is based on a mechanism dependent on Fc*γ*- pathway [35].

Classical immunoreceptors, such as BCRs, T cell receptors (TCRs), and various FcRs, facilitate the adaptive detection of both self and foreign antigens, either through direct or indirect mechanisms [36]. All the aforementioned receptors interact with transmembrane proteins that feature cytoplasmic domains incorporating immunoreceptor tyrosine-based activation motifs (ITAMs). Dual phosphorylation of ITAMs leads to the activation of Syk, which ultimately drives downstream signaling pathways. More specifically, Syk modulates various biological processes depending on the cell type and the receptor interacting with it, including mast cell and basophil degranulation, proinflammatory cytokine production by macrophages, monocytes, and dendritic cells, as well as antibody production by B cells in response to specific antigens [29, 37]. Moreover, the activation of Fc*γ*Rs on myeloid cells initiates a Syk-dependent signaling pathway, which facilitates the internalization of IgG-opsonized antigens, cells, or pathogens via phagocytosis [29]. IgG autoantibodies that bind to self-antigens on cells, such as platelets and red blood cells, can form immune complexes that subsequently activate Fc*γ*Rs. This process contributes to the pathogenesis of autoimmune disorders, including ITP. Syk's diverse functional activity depends on multiple phosphorylation sites. Several phosphorylated residues of Syk serve as essential structural elements for recruiting downstream signaling proteins, thereby coordinating cellular responses [37].

BTK is involved in the signaling pathway of Fc*γ*Rs, BCR signaling, Toll-like receptor (TLR), B-cell activating factor (BAFF) signaling, and chemokine receptor signaling [38, 39]. The interaction of specific antigens with the BCR leads to the phosphorylation of ITAMs through Lck/Yes-related novel Tyk (LYN), a member of the SRC family of nonreceptor tyrosine kinases [40]. This sequence of events recruits Syk, a nonreceptor protein Tyk which phosphorylates BLNK, a B-cell linker protein that functions as a scaffold to facilitate the interaction of BTK and phospholipase C-*γ*2 (PLC*γ*2). Ultimately, phosphorylated PLC*γ*2 followed by activation of the nuclear factor of activated T cells (NFAT), nuclear factor-*κ*B (NF-*κ*B), and mitogen-activated protein kinase (MAPK) pathways, stimulate cell proliferation and differentiation, and production of antibody and cytokine [40]. Moreover, BAFF/BAFF-R interaction also prevents Syk inhibition, as well as promotes BTK activation, which plays a crucial role in B-cell survival, activation, and Ig production [39].

Both Syk and BTK have been identified as critical regulators for Fc*γ*-mediated platelet destruction and Fc*γ* -receptor-mediated signaling [34, 41]. It has been established that, in murine models of ITP, antiplatelet antibodies are necessary, but not sufficient for disease induction; functional phagocytic Fc*γ*Rs are also critical [42]. In a murine model of the inflammatory response, severe thrombocytopenia occurred in ITP mice models injected with an antiplatelet antibody (mAB 6A6); however, FcR*γ*-deficient mice did not develop anemia or thrombocytopenia, indicating that FcR signaling is necessary for murine ITP development [29, 43].

Since BTK is present in numerous hematopoietic stem cells and has a crucial role in both the adaptive and innate immune responses, it is a key therapeutic target in autoimmune disorders [44, 45]. BTK inhibition was found to lead to positive outcomes in some autoimmune diseases, including ITP [40]. For instance, mice lacking BTK were found to be protected against SLE and autoimmune arthritis [46]. It has also been illustrated that orelabrutinib prescription in mice was associated with a significant elevation in platelet count [41]. Furthermore, another study exhibited that pretreatment with rilzabrutinib, a BTK inhibitor, in mouse models of ITP induced by IgG antibodies resulted in a dose-dependent and rapid diminution in platelet destruction, effectively preserving the mice from ITP [44].

Currently, Syk inhibitors are under investigation in clinical trials for immune-mediated diseases, including ITP, due to the important role of Syk-mediated opsonized platelet phagocytosis in ITP pathogenesis [29]. Syk inhibition has demonstrated effectiveness in mitigating antibody-driven pathology across various rodent disease models, such as vasculitis, arthritis, autoimmune hemolytic anemia (AIHA), glomerulonephritis, and acute lung injury [29]. Additionally, Syk inhibition has been shown to improve thrombocytopenia and increase platelet and red blood cell counts in a murine antibody-mediated model of ITP [47]. All these findings emphasize the role of BTK and Syk as crucial factors in ITP pathogenesis and highlight the potential of targeting these pathways as a therapeutic option.

### 3.2. Syk Inhibitors

Fostamatinib is the only Syk inhibitor that has received FDA approval for treating refractory ITP cases [48]. Fostamatinib inhibits the phosphorylation of Syk substrates, thereby preventing the activation of T cells and B cells [49, 50]. This leads to the suppression of antibody-mediated and immune complex-mediated pathways, which are critical mechanisms of platelet destruction in ITP. A meta-analysis conducted on randomized clinical trials investigating fostamatinib in refractory ITP patients demonstrated that this medication was associated with superior efficacy in comparison with conventional therapy alone in terms of obtaining stable platelet response by week 24 as well as platelet count ≥50,000/µL at weeks 12 and week 24 [51]. Moreover, adverse events reported in the meta-analysis, as mentioned earlier, included diarrhea, hypertension, and abnormal liver function tests. Overall, most adverse events with fostamatinib therapy were mild to moderate, highlighting the suitable safety profile of fostamatinib. Notably, ITP cases are generally at a greater risk of thromboembolic events; however, patients treated with fostamatinib demonstrated a lower incidence rate of thromboembolic events compared to older therapies [52]. Fostamatinib decreases the incidence of thromboembolic events by inhibiting all three major Syk-driven signaling contributing to pathogenic platelet aggregation, including FcyRIIA gamma globulin receptor, glycoprotein VI (GPVI) receptor, and C-type lectin-like II receptor CLEC-2. It is noteworthy that fostamatinib does not affect other platelet receptors involved in hemostasis, thereby preventing unwanted bleeding [53, 54].

Sovleplenib and cevidoplenib are two novel Syk inhibitors under investigation for ITP management. Sovleplenib is a small molecule, potent, and selective oral Syk inhibitor that has been studied in one randomized, double-blind, placebo-controlled, Phase 3 study and one randomized, multicenter, open-label, Phase 2 study. In a Phase 1b/2 study performed by Liu et al. [18], 45 cases with primary ITP with at least 6 months duration that failed or relapsed after previous first-line treatment were treated with either sovleplenib or placebo over two phases: dose-escalation and dose-expansion phases, each with an 8-week, double-blind, placebo-controlled period, followed by a 16-week, open-label period with sovleplenib. During the dose-escalation phase, 33 cases of patients were divided into five groups based on the dosage of sovleplenib: Group1 (G1) received a placebo (*n* = 9), while G2 (*n* = 6), G3 (*n* = 6), G4 (*n* = 6), and G5 (*n* = 6) received 100, 200, 300, and 400 mg, respectively. Twelve subjects assigned to the dose-expansion phase were recruited in the dose-expansion phase, of which 10 patients received sovleplenib and two underwent a placebo. A response was characterized by a platelet count increase to 30 × 10^9^/L or higher, with at least a twofold rise from baseline, observed consistently over two consecutive visits within the first 8 weeks of the trial period, without the administration of rescue therapy. The most significant rise in platelet count was noted in the sovleplenib 300 mg group, with increased platelet levels detectable from week 1 among responders. The number of patients who achieved the primary efficacy endpoint with sovleplenib was three in the 100 mg group (50%), three in the 200 mg group (50%), 10 in the 300 mg group (63%), and two in the 400 mg group (33%), whereas only one patient in the placebo group (9%) met this endpoint. Moreover, the sovleplenib 300 mg group exhibited the highest overall response rate during the 0–8 weeks period, followed by the 100 mg group. Additionally, the quickest response to treatment occurred in the sovleplenib 300 mg group with a median time of 1.1 weeks. Phase 2 of the study was conducted with oral sovleplenib at a dosage of 300 mg for a duration of 16 weeks. In the 300 mg sovleplenib group, the overall response rate was 80%, comprising 16 of 20 participants who either received continuous sovleplenib or transitioned from placebo. Among those on continuous 300 mg sovleplenib, the durable response rate was 31%, while it was 75% for the four participants who crossed over from placebo to sovleplenib between weeks 0 and 24. This higher response rate may have occurred because the crossover group lacked prior exposure to the drug, potentially enabling them for a more effective response when they began treatment. Moreover, the subjects who transitioned from placebo to sovleplenib may have had relatively higher platelet counts and less resistant disease at the time they received the treatment. The safety profile of sovleplenib in patients with primary ITP was generally consistent across doses ranging from 100 to 400 mg. This safety profile, along with the activity and pharmacokinetic findings during dose escalation, led the safety review committee to recommend 300 mg of sovleplenib once daily as the Phase 2 dose. Most adverse events were classified as mild to moderate in severity and included elevated aspartate aminotransferase (29%), alanine aminotransferase (27%), and blood lactate dehydrogenase (22%). There were no treatment-emergent adverse events (TEAEs) that led to treatment discontinuation or death. Furthermore, none of the patients treated with sovleplenib experienced thrombotic events associated with the medication.

Hu et al. [17] conducted a study to assess the efficacy and safety of sovleplenib in patients with chronic primary ITP who had a median of four prior treatment lines, including agents such as anti-CD20 antibodies and TPO or TPO-RAs. The study was conducted in two phases: in the double-blind phase of the study, oral sovleplenib with a dosage of 300 mg was compared with a matched placebo in 188 adults for a duration of 24 weeks. As a result, the study achieved its primary endpoint, showing that the durable response (a platelet count of ≥50 × 10^9^/L on at least four of six scheduled visits between weeks 14 and 24) rate was significantly greater in the sovleplenib group compared to the placebo group. Moreover, at all postbaseline visits, the median platelet counts were higher in the sovleplenib group than in the placebo group. Sovleplenib also demonstrated a significantly higher overall response rate compared to placebo during both the 0–12 week and 0–24 weeks periods. A post hoc analysis also showed that the proportion of patients with a platelet count of 30 × 10^9^/L or more was significantly greater in the sovleplenib group than in the placebo group across both time intervals. During the double-blind phase, patients receiving sovleplenib required substantially less rescue medication than those on placebo. Additionally, more patients in the sovleplenib group either reduced or discontinued their baseline concomitant anti-ITP treatment (*n* = 11%, 27%) compared to the placebo group (*n* = 2%, 10%). The ability of the treatment to sustain stable platelet levels likely contributed to this outcome, as patients receiving sovleplenib were less prone to experiencing rapid platelet count declines that would require rescue therapy. This indicates that sovleplenib not only provided direct benefits in increasing platelet counts but also exerted a broader positive effect by reducing the need for additional medications, potentially improving the burden of long-term therapy. Additionally, sovleplenib induced a faster response, with a median time to response of 8 days, compared to 30 days for a placebo. Notably, sovleplenib demonstrated a more rapid onset of action, with a median time to response of 8 days, whereas fostamatinib [55] (with biweekly visits) and TPO [56] or TPO-RAs [57] (with weekly visits) typically required more than 14 days to elicit a response. The incidence of bleeding, as measured by the WHO bleeding score, showed a decreasing trend from baseline to week 24 in the sovleplenib group. Regarding safety, most adverse events were mild to moderate, and sovleplenib was tolerable. Notably, 125 out of 126 cases in both groups experienced TEAEs of any grade. Moreover, four patients (3%) in the sovleplenib group experienced TEAEs that led to discontinuation, including increased blood creatinine (Grade 1), weight gain (Grade 1), and hemorrhage (Grade 3), each affecting one patient, while another patient experienced elevated alanine aminotransferase (Grade 3) and aspartate aminotransferase (Grade 2). In the open-label phase of the investigations, cases of both groups who did not respond in the first 12 weeks of the study could terminate the double-blinded phase and enroll in the open-label study receiving sovleplenib 300 mg once daily, which is still ongoing (NCT05029635).

Cevidoplenib is another novel Syk inhibitor that is under investigation in patients suffering from persistent or chronic ITP who failed or relapsed after at least one prior therapy (NCT04056195) [58]. Cevidoplenib is a selective Syk inhibitor engineered to target downstream signaling of B-cell and FcRs without affecting T-cell functions or other pathways involved in cytokine-mediated inflammation. The preliminary results of this Phase 2 trial have illustrated that the platelet response, defined as a count of at least 30,000/µL and doubled their baseline total at any point during the treatment period without the use of rescue medication, occurred in 64% of cases underwent 400 mg of cevidoplenib compared to 33% of subjects who received placebo.

### 3.3. BTK Inhibitors

As of yet, two BTK inhibitors, rilzabrutinib and orelabrutinib, have been examined in ITP management.

Rilzabrutinib is an orally available, selective, and reversible covalent inhibitor targeting BTK, particularly developed for immune-mediated disease management [30]. Its therapeutic action operates via two primary mechanisms: (1) suppression of B-cell activation and (2) inhibition of Fc*γ*R-driven phagocytosis of antibody-coated cells in the spleen and liver. Rilzabrutinib has depicted favorable outcomes in two clinical trials published to date. In the open-label, dose-finding Phase 1/2 clinical trial carried out by Kuter et al. [31], the efficacy and safety of rilzabrutinib were investigated in 60 patients with chronic or refractory ITP who did not respond to prior medications. Patients received rilzabrutinib at one of the following dosing regimens: 200 mg once daily, 400 mg once daily, 300 mg twice daily, or 400 mg twice daily for a duration of 24 weeks. Consequently, 40% of participants achieved the primary endpoint, which was described as a platelet count of ≥50 × 10^9^/L with an increase of at least 20 × 10^9^/L from the pretreatment baseline. Distinctively, higher doses of rilzabrutinib were associated with superior outcomes, with 18 out of 45 patients who underwent rilzabrutinib at the highest dose achieving the primary endpoint. Key therapeutic objectives for individuals with ITP include achieving a rapid elevation in platelet count to levels sufficient to prevent bleeding and maintaining this response over time. In this regard, rilzabrutinib therapy resulted in rapid platelet responses, with clinically significant platelet increases observed in 40% of patients, with the median time to obtain a platelet count of at least 50 × 10^9^/L was 11.5 days. Considering safety, all TEAEs were mild to moderate, and the investigation reported no treatment-related bleeding, severe adverse events, infections, deaths, or typical signs and symptoms commonly associated with other irreversible BTK inhibitors.

In the long-term extension of the prior open-label Phase 1/2 clinical trial, 16 patients who responded during the initial 24-week investigation continued rilzabrutinib treatment at a dosage of 400 mg twice daily [32]. After a median treatment duration of 1.3 years, 11 out of 16 subjects remained on rilzabrutinib therapy, with 93% of these patients achieving a platelet count of ≥50 × 10^9^/L in over half of their monthly assessments. Moreover, five out of 11 subjects who were prescribed concomitant therapy for their ITP could cease their concomitant medications. With respect to safety, three cases experienced mild to moderate TEAEs, including Grade 2 upper respiratory tract infection, rhinorrhea, vulvovaginal dryness, and Grade 1 cough and diarrhea. Moreover, serious AEs were reported in three patients; all events were determined to be unrelated to the study treatment. Importantly, rilzabrutinib was not associated with bleeding, arrhythmia, or other AEs typically linked to nonselective BTK inhibitors. Furthermore, despite a very low rate of bleeding at the start of the study, bleeding scores improved even further with continued rilzabrutinib treatment in the long-term extension. Compared to other treatments, in an open-label extension study, romiplostim treatment resulted in achieving a platelet response of ≥50 × 10^9^/L at least once in 95% of patients, with the response being sustained for a median of 92% of visits [59]. However, rescue medication was required in 33% of patients, and TEAEs occurred in 35% of patients, with 8% classified as serious. Notably, bleeding events were observed in 57% of patients, while thrombotic events occurred in 6.5%. In another clinical trial conducted on eltrombopag [60], a remarkable proportion, 32% of patients, experienced Grade ≥3 AEs (6% had thromboembolic events) despite clinical effectiveness. Currently, rilzabrutinib at a dosage of 400 mg twice daily is under investigation in adolescents and adults with ITP in a multicenter, double-blind, and placebo-controlled Phase III study (LUNA 3, NCT04562766) [30]. The upcoming results of this study are still awaited.

Orelabrutinib, a next-generation BTK inhibitor, offers enhanced selectivity and sustained inhibition of BTK while minimizing off-target impacts [61]. In a Phase 2 clinical trial carried out by Yan et al. [33], 33 patients with chronic or persistent ITP were assessed for the effectiveness and tolerability of Orelabrutinib therapy. Patients were divided into two groups based on the Orelabrutinib dosage (G1:30 mg and G2:50 mg). Out of the 18 patients in the 30 mg group, 13 had their dosage increased to 50 mg by week 4. The primary endpoint of platelet response was reached by 12 patients (36%): six out of 15 patients in G2 and six out of 18 patients in G1, including two of the 13 patients who transitioned from G1 to G2. The sustained response rate was 27% in G2 and 33% in G1, including two of the 13 patients who transitioned from G1 to G2. Of the 12 patients who reached the primary endpoint, 83% maintained a sustained response. G2 achieved a notably quicker median time to an initial platelet count increase than G1. G2 had the lowest rate of patients using concomitant rescue therapy, with six out of 15 patients (40%) reported to have done so. Notably, in cases that had previously shown a response to glucocorticoids or IVIG, the primary endpoint response rate in the 50 mg group was reported in 75% of patients. This finding highlighted that orelabrutinib may demonstrate elevated efficacy in individuals who have responded to prior first-line treatments. The efficacy results of orelabrutinib were similar to those of rilzabrutinib in a Phase 1/2 study, which showed primary and sustained response rates of 40% and 28%, respectively [32]. A total of 28 patients (85%) reported side effects, the majority of which were mild (Grades 1 or 2), and only 24% were deemed treatment-related. One patient in G1 had a TRAE that required treatment cessation due to Grade 2 pneumonia. No serious or Grade 3 TRAEs or fatalities were reported.

## 4. Conclusions

The treatment landscape for ITP remains challenging, particularly for patients who do not respond to conventional therapies, resulting in a significant treatment burden. The pathogenesis of ITP involves intricate immunological mechanisms, with Syk and BTK playing central roles in FcR-mediated pathways, which drive both platelet destruction and autoantibody production. Advances in targeted therapies have highlighted the potential of Syk and BTK inhibitors in addressing these pathogenic mechanisms. Syk inhibitors like fostamatinib have shown efficacy in increasing platelet counts and maintaining stability in refractory ITP patients. Emerging BTK inhibitors, including rilzabrutinib and orelabrutinib, also demonstrate promise by attenuating autoimmune responses and reducing FcR-driven platelet phagocytosis. As clinical data on these inhibitors continue to grow, these therapies may redefine treatment paradigms for ITP, offering new options with potentially lower adverse event profiles and improved patient outcomes. Further research is needed to optimize these treatments and to assess their long-term safety and efficacy across diverse patient populations.

## Figures and Tables

**Table 1:** Characteristics of eligible studies utilizing Syk inhibitors in the treatment of ITP.

Study ID (Author, year)	Study design	Sample size	Age	Gender	Disease type	Disease duration	Baseline characteristics	Previous treatment(s)	Treatment(s) of the study	Treatment details	Concurrent treatment(s)	Outcome measurement (s)	Clinical efficacy	Safety
Liu, 2023 [18]	Randomized, double-blind, placebo-controlled, Phase 1b/2 study	45 dose escalation phase: 33 dose expansion phase: 12	18–75	F:60%	Chronic primary ITP	More than 6 months	Two consecutive platelet counts (interval of >24 h) of less than 30 × 10^9^/L, hemoglobin 90 g/L or higher, white blood cells higher than 2.5 × 10^9^/L, median platelet count at baseline of 6.5 × 10^9^/L in sovleplenib groups versus 19.0 × 10^9^/L in the placebo group and platelet count of lower than 15 × 10^9^/L in 27 (79%) of 34 versus 4 (36%) of 11 patients	Glucocorticoids, previous thrombopoietin or TP-RAs, traditional Chinese medicine, IVIG, platelet transfusion, cyclosporine, Anti-CD20, danazol, IL-11, azathioprine, vinblastine, neonatal Fc receptor clinical study, sirolimus, mycophenolate mofetil, previous splenectomy, systemic corticosteroids	Syk inhibitor	Placebo (*n* = 11) Sovleplenib orally once daily: 100 mg (*n* = 6), 200 mg (*n* = 6), 300 mg (*n* = 16; 6 patients from the dose-escalation phase and 10 from the dose-expansion phase), and 400 mg (*n* = 6) 8 weeks of double-blind period (*n* = 45), 16 weeks of open-label period (*n* = 36)	Concomitant anti-ITP therapy (without rescue therapy)	The proportion of patients whose platelet count reached 30 × 10^9^ platelets per L or higher and was double that of the baseline at two consecutive visits during 0–8 weeks without rescue therapy, the proportion of patients with at least one platelet count of 50 × 10^9^ platelets per L or higher (overall response rate), bleeding score at each visit, the proportion of patients who received rescue treatment	The median duration of treatment exposure of 55 days in the placebo groups and 56 days in the sovleplenib groups, during weeks 0-8, the greatest increase of platelet count in the sovleplenib 300 mg group and increased platelet counts were observed from week 1 for responders, higher median platelet count (higher than 50 × 10^9^ platelets per L at week 1 [day 8]) in the sovleplenib group than the placebo group at most scheduled study visits during the double-blind period, the proportion of patients who met the main efficacy endpoint with sovleplenib: 3 (50%) in the 100 mg group, 3 (50%) in the 200 mg group, 10 (63%) in the 300 mg group, and 2 (33%) in the 400 mg group compared with 1 (9%) in the placebo group, the highest overall response rate during 0–8 weeks in the sovleplenib 300 mg group (11 [69%] of 16 patients), followed by the 100 mg group (3 [50%] of 6)	Serious AEs in 4 patients (two [18%] in the placebo group and two [6%] in the sovleplenib 100 mg group), which were considered not related to the study drug most frequently reported sovleplenib-related AEs: increased AST, ALT, and blood lactate dehydrogenase

Hu, 2024 [17]	Randomized, double-blind, placebo-controlled, Phase 3 study	188 G1: 126 G2: 62	18–75	F: 66%	Chronic ITP	At least more than a year	Eastern Cooperative Oncology Group performance status of 0–1, had failed or relapsed after receiving at least one treatment, a WHO bleeding score of 0–1, a mean platelet count of less than 30 × 10^9^/L at three visits (platelet count of ≤35 × 10^9^/L at any of them) within 3 months before randomization, hemoglobin level at 100 g/L or more, and a neutrophil count of more than 1.5 × 10^9^/L	Thrombopoietin or TPO-RA, glucocorticoid, traditional Chinese medicine, IVIG, platelet transfusion, danazol/stanozolol, caffeic acid, Anti-CD20 antibody, ciclosporin, IL-11, splenectomy	Syk inhibitors	G1: sovleplenib 300 mg once daily (*n* = 126) for 24 weeks G2: placebo (*n* = 62) for 24 weeks Upon completion of the first 12 weeks of treatment in the double-blind period, patients who did not respond (platelet count <50 × 10^9^/L) to study treatment in both groups were allowed to end the double-blind treatment early and receive open-label treatment of sovleplenib 300 mg once daily	One of the following treatments for each patient: a stable dose of prednisone, danazol, or immunosuppressants (one of azathioprine, cyclosporine A, and mycophenolate mofetil). Dose reduction of concomitant treatment for primary ITP was allowed during the treatment period, when a platelet count of 100 × 10^9^/L or more lasted for 4 weeks or a platelet count of 250 × 10^9^/L or more lasted for 2 weeks	Primary endpoint: durable response rate. Secondary endpoints: overall response rate in 0–12 weeks and 0–24 weeks; the proportion of patients with a platelet count of 30 × 10^9^/L or more plus an increase of 20 × 10^9^/L or more from baseline in 0–12 weeks and 0–24 weeks for the patients with a baseline platelet count below 15 × 10^9^/L; time to response; bleeding incidence and severity per WHO bleeding score in 0–12 weeks and 0–24 weeks	The durable response rate of 48% in G1 compared with zero in G2, median time to response of 8 days with sovleplenib compared with 30 days with placebo, higher median platelet counts in G1 (50–90 × 10^9^/L) than in G2 (<30 × 10^9^/L) at all postbaseline visits, a significantly higher overall response rate in G1 compared with G2 at 0–12 weeks and 0–24 weeks, significantly higher proportion of patients with a platelet count of 30 × 10^9^/L or more in the G1 than in the G2 at 0–12 weeks and 0–24 weeks, a significant reduction in the use of rescue medication in G1 compared to, a decreased trend from baseline to week 24 in the bleeding incidence per WHO bleeding score in the G1, significantly lower mean of the WHO bleeding score in G1 compared to G2 at 0–12 weeks and 0–24 weeks	The most common TEAEs of Grade 3 or higher: platelet count decreased (7% [9/126] vs. 10% [6/62]), neutrophil count decreased (3% [4/126] versus 0% [0/62]), and hypertension (3% [4/126] versus 0% [0/62] treatment-related serious TEAEs: pain in the extremity (Grade 2) and rash maculo-papular (Grade 1) in one patient and hypertension (Grade 3) in the other patient in G1, cerebral hemorrhage (Grade 3) in one patient in G2 TEAEs leading to discontinuation in 4 (3%) patients in G2: blood creatinine increased (Grade 1), weight increased (Grade 1), and hemorrhage (Grade 3), each in one patient; ALT increased (Grade 3) and AST increased (Grade 2) in the same patient

Abbreviations: ALT, alanine aminotransferase; AST, aspartate aminotransferase; BTK, Bruton's tyrosine kinase; F, female; Fc, fragment crystallizable; G, group; ITP, immune thrombocytopenia; IVIG, intravenous immunoglobulin; Syk, spleen tyrosine kinase; TEAE, treatment-emergent adverse event; TPO-RA, thrombopoietin Receptor Agonist; Tyk, tyrosine kinase; WHO, World Health Organization.

**Table 2:** Characteristics of eligible studies utilizing Bruton's tyrosine kinase (BTK) inhibitors in the treatment of ITP.

Study ID (Author, year)	Study design	Sample size	Age	Gender	Disease type	Disease duration	Baseline characteristics	Previous treatment(s)	Treatment(s) of the study	Treatment details	Concurrent treatment(s)	Outcome measurement (s)	Clinical efficacy	Safety
Kuter, 2022 [31]	Open-label, dose-finding, Phase 1–2 clinical trial	60	50	F: 57%	Refractory chronic ITP	6.3 years	Platelet counts of less than the median baseline platelet counts of 15 × 10^9^/L on two occasions no less than 7 days apart within the 15 days before trial entry with a response to at least one previous therapy for ITP (including splenectomy) but to not have had a response to the previous or concomitant therapy maintained at baseline	Most common previous therapies: glucocorticoids (92%), TPO-RAs (58%), intravenous immune globulin (43%), rituximab (40%), and splenectomy (25%)	BTK inhibitor	Rilzabrutinib The initial dose could be 200 mg once daily, 400 mg once daily, 300 mg twice daily (i.e., 600 mg per day), or 400 mg twice daily (i.e., 800 mg per day; the highest dose). Intrapatient dose escalation was allowed every 28 days, according to the investigator's judgment, to improve response	Stable concomitant therapy with a glucocorticoid or TPO-RA with no more than a 10% change in the dose within the 2 weeks before the initiation of rilzabrutinib	Primary endpoint: platelet response (at least two consecutive platelet counts of at least 50 × 10^3^ per cubic millimeter and an increase from baseline of at least 20 × 10^3^ per cubic millimeter without the use of rescue medication) Secondary endpoints: the percentage of weeks with a platelet count of at least 50 × 10^3^ per cubic millimeter, the percentage of patients who had a platelet count of at least 50 × 10^3^ per cubic millimeter at four or more of the final eight platelet counts, the number of weeks with a platelet count of at least 50 × 10^3^ per cubic millimeter, the number of weeks with a platelet count of at least 30 × 10^3^ per cubic millimeter	The primary endpoint of platelet response achievement in 24 patients (40%), 1 of 9 patients (11%) met the primary endpoint at a dose of 200 mg once daily, 2 of 8 (25%) at a dose of 400 mg once daily, 4 of 12 (33%) at a dose of 300 mg twice daily, and 20 of 52 (38%) at a dose of 400 mg twice daily (the highest dose). Of the 45 patients who had started rilzabrutinib at the highest dose, 18 (40%) met the primary endpoint of platelet response, 17 of 60 patients (28%) had a platelet count of at least 50 × 10^3^ per cubic millimeter at four or more of the final eight platelet counts, among patients with a response, 24 in the overall trial population had a platelet count of at least 50 × 10^3^ per cubic millimeter maintained for a median of 16 weeks	At least one TEAE in 31 patients (52%), the most common TEAEs of any grade: diarrhea (32%), nausea (30%), and fatigue (10%) No treatment-related bleeding or thrombotic events of Grade 2 or higher, treatment-related adverse events of Grade 3 or higher, and serious adverse events, no other signs or symptoms of adverse events that have been typically associated with BTK inhibitors

Kuter, 2024 [32]	Long-term extension of open-label, dose-finding, Phase 1–2 clinical trial	16	49	F: 56%	Refractory chronic ITP	4.3 years	Median platelet count was 87 × 10^9^/L	Splenectomy (19%)	BTK inhibitor	Rilzabrutinib 400 mg twice	Concomitant ITP medication in 11 cases (7 patients had CS, 2 TPO-RA, and 2 both concomitant CS and TPO-RA)	The primary efficacy endpoint: ≥2 consecutive platelet counts (separated by ≥5 days) of ≥50 × 10^9^/L and an increase from baseline of ≥20 × 10^9^/L without the use of rescue medication for ITP in the previous 4 weeks before the latest elevated platelet count	The median percentages of weeks with a platelet count of ≥30 × 10^9^/L, ≥30 × 10^9^/L along with ≥20 × 10^9^/L over baseline, and ≥50 × 10^9^/L were 100%, 97%, and 88%, respectively. Of the 14 patients who received treatment with ≥1 monthly evaluation for >6 months on long-term extension, the median percentage of visits with a platelet response of ≥50 × 10^9^/L was 92%. 5 of 11 (45%) patients receiving concomitant ITP therapy were able to stop using any concomitant ITP medication (CS, *n* = 2; TPO-RA, *n* = 1; CS and TPO-RA, *n* = 2).	≥1 any-cause AEs in 13 patients (81%), with 3 patients (19%) experiencing grade ≥3 AEs TEAEs in 3 patients (19%; mild to moderate): Grade 1 or 2 and transient; Grade 2 upper respiratory tract infection, rhinorrhea, vulvovaginal dryness, and Grade 1 cough and diarrhea Serious AEs in three patients (unrelated to the study): Grade 4 COVID-19 interstitial pneumonia, Grade 4 thrombocytopenia, and Grade 3 pneumonia/Grade 3 pulmonary embolism

Yan, 2024 [33]	Randomized, multicenter, open-label, Phase 2 study	33	18–80	NA	Persistent or chronic ITP	9 years	Mean platelet count of ≤35 × 10^9^/L on 2 occasions at least one day apart and a measured platelet count of ≤35 × 10^9^/L, who had failed at least one prior line of standard therapy or failed to tolerate a standard therapy	Corticosteroids, IVIG, others (not mentioned)	BTK inhibitors	G1: orelabrutinib 30 mg once daily for 24 weeks, which could increase the dose to 50 mg if platelet counts were <50 × 10^9^/L week 4 without other safety concerns (*n* = 18) G2: 50 mg once daily for 24 weeks	No concurrent treatment	The proportion of patients achieving platelet counts of ≥50 × 10^9^/L for at least two consecutive weeks (without rescue medication in the prior 4 weeks), 36-Item Short Form Health Survey for assessing quality of life and physical well-being	A total of 10 patients (30%) achieved a sustained response of measured platelet counts of ≥50 × 10^9^/L in 4 or more of the final 6 visits between 14 and 24 weeks (sustained response rate of 27% in the 50 mg group and 33% in the 30 mg group) Orelabrutinib treatment improved patients' quality of life, with an average increase of 21.2 points in physical well-being and 10.3 points in emotional well-being using the 36-Item Short Form Health Survey (SF-36) scores measured at week 24. Patients in the 50 mg group experienced reduced bleeding scores compared to baseline. effective increase in platelet counts in patients with primary ITP, especially in those who previously responded to glucocorticoids or IVIG with once-daily oral administration of orelabrutinib	A total of 28 patients (85%) experienced side effects; most were mild (Grades 1 or 2), with only 24% considered treatment-related

Abbreviations: ALT, alanine aminotransferase; AST, aspartate aminotransferase; BTK, Bruton's tyrosine kinase; F, female; Fc, fragment crystallizable; G, group; ITP, immune thrombocytopenia; IVIG, intravenous immunoglobulin; Syk, spleen tyrosine kinase; TEAE, treatment-emergent adverse event; TPO-RA, thrombopoietin receptor agonist; Tyk, tyrosine kinase; WHO, World Health Organization.

## Data Availability

The data that supports the findings of this study are available in the text and tables.

## Nomenclature

BAFF: B-cell activating factor
BCR: B-cell receptor
BTK: Bruton's tyrosine kinase
Fc*γ*R: Fc*γ* receptor
G: Group
GPVI: Glycoprotein VI
ITAM: Immunoreceptor tyrosine-based activation motif
ITP: Immune thrombocytopenia
IVIG: Intravenous immunoglobulin
MAPK: Mitogen-activated protein kinase
NFAT: Nuclear factor of activated T cells
NF-*κ*B: Nuclear factor-*κ*B
PLC*γ*2: Phospholipase C-*γ*2
Syk: Spleen tyrosine kinase
TCR: T cell receptor
TEAE: Treatment-emergent adverse event
TLR: Toll-like receptor
TPO-RA: Thrombopoietin receptor agonist
Tyk: Tyrosine kinase.
